# Grain boundary anisotropy on nano-polycrystalline magnetic thin films

**DOI:** 10.1038/s41598-020-61979-z

**Published:** 2020-03-19

**Authors:** Jose D. Agudelo-Giraldo, Elisabeth Restrepo-Parra, Johans Restrepo

**Affiliations:** 1 PCM Computational Applications, Universidad Nacional de Colombia Sede Manizales, Manizales, Colombia; 2 Departamento de Física y Matemática, Universidad Autónoma de Manizales, Manizales, Colombia; 3 Grupo de Magnetismo y Simulación G+, Instituto de Física, Universidad de Antioquia, A.A. 1226 Medellín, Colombia

**Keywords:** Materials science, Nanoscience and technology, Physics

## Abstract

Grain boundaries in polycrystalline thin films with crystallite sizes at nanoscale presents regions characterized by a high degree of local structural disorder. As a consequence, great values of the associated local anisotropies are expected. On this regard, a systematic investigation of the effect of the grain boundary anisotropy on the magnetic properties in such type of nanostructured systems is addressed. For developing this work, a standard Monte Carlo simulation in the framework of classical Heisenberg spins was carried out, with a Hamiltonian involving exchange couplings, dipolar interactions, Zeeman interaction and contributions of cubic magneto-crystalline anisotropy. A quantification of local structural disorder was considered. Results revealed that i) by keeping the same number of grains, different organizations give rise to different spontaneous magnetizations, ii) the critical exponent of the magnetization differs of pure models, which is attributed to the complexity of the lattice and consistent with a distribution of critical temperatures, iii) Boundary anisotropy varies with temperature and its strength are determinant factors for blocking temperatures, and iv) Boundary anisotropy inside in the hysteretic properties where coercive field variations are observed.

## Introduction

New technological components used to confine and guide magnetic fields, such as inductive sensors, flexible antennas and magnetic cores devices, have been developed to take advantage of nano-magnetic properties. The nanostructured systems composed by nanoparticles and ultra-thin films with nano-grains structure had taken great importance by the new and intrigant phenomenally. One of the well-known properties of nanoparticles systems is the fact that coercive field decreases strongly with diminution of the mean diameter in the single-domain regime per particle. Coercivity can goes strictly to zero in a superparamagnetic state (SPM)^1^. However, SPM is not clear in nanograins, the reason is nanograins do not loss completely the connectivity between them. In this case, domain structure can be understood in terms of the magnetic moments fluctuations per nanograin or a low number of them around the superparamagnetic limit (SPL)^2^. Different models have been proposed to quantify the magnetic couplings between grains^3–6^. The random anisotropy model (RAM) proposed by Herzer is one of the most relevant^5^. This model had contributed to explain some of the properties at micrometric scale. However, new models are necessary at a more reduced scale where the ratio between atoms in grain boundaries and atoms in the core grains increases considerably.

This work presents results of a Monte Carlo simulation of magnetic polycrystalline samples. The study is based on the fact that boundaries alter the magnetic behaviour. The parameters of the different magnetic contributions were adjusted to experimental values. Typically, the magneto-crystalline anisotropy, the surface anisotropy and the dipolar interaction are in µeV range. Therefore, the difference is well stablished respect to exchange interaction which is in the meV range. In case of boundary anisotropy, this might have different orders of magnitude. When both, exchange and boundary anisotropy are in a direct competition, a loss of length correlation in boundaries is reflected in magnetic properties. In particular, the blocking temperature studies give indications of the factors for which the magnetic domains fluctuant independently by thermal effect and the hysteresis loop studies give indications the effect of the intensity of the boundary anisotropy upon the loss of magnetic coupling between grains.

## Model description

**Figure 1:**
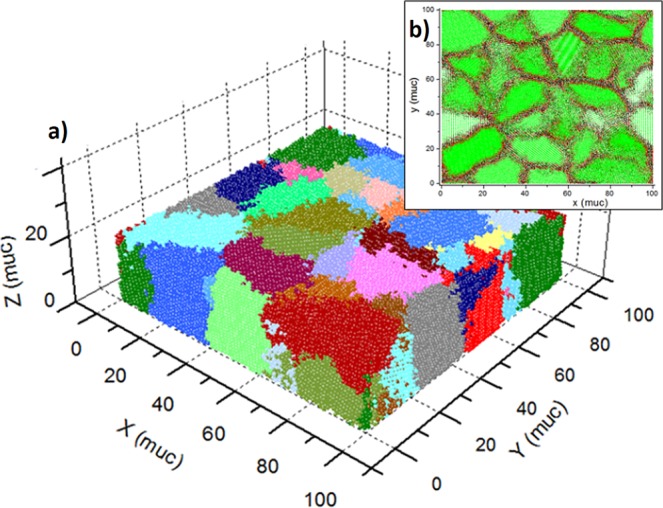
(**a**) Polycrystalline simulated sample with $L=100muc$ $d=20muc$ and $({N}_{g})=30$. Periodic boundar*y* conditions along *x*-*y* plane can be identified looking at the colour distribution of the grains. (**b**) Surface view highlighting grain boundaries.

Polycrystalline thin film samples were simulated by considering a simple cubic (SC) lattice structure. Periodic boundary conditions (PBC) were implemented for giving continuity to the granular growth along the *x-y* plane, while free boundary conditions along *z* axis were taken to account. A typical simulated sample is shown in Fig. 1. The procedure for sample construction was detailed in a previous work where a process of structural relaxation in boundaries was taken into account^7^. Linear dimensions are given in magnetic unit cells ($muc$) according to magnetic moments positions into the crystal lattice. Sample dimension was set at $L=100muc$ in *x-y* plane with a thickness of $d=20muc$. It was employed 2 × 10^5^ atoms in 10 different samples where number of grains $({N}_{g})$ was keep at 30.

The code was compiled using gFortran and OpenMP software. A parallelized Monte Carlo method using 10 cores with shared memory access was implemented. In such a way that sample was divided into 10 × 10 × 2 cubic cells of the same volume, each of one was assigned to single computational core. The number of Monte Carlo steps (MCS) was fixed at 3.2 × 10^4^ per cell, which was enough for thermalization purposes according to energy relaxation. Magnetization was recorded by following a cooling process where temperature ranged from 400 K down to 2 K every 0.25 K.

The Hamiltonian describing the interactions in the system reads as follows:

1 $$ {\mathcal H} ={ {\mathcal H} }_{exc}+{ {\mathcal H} }_{an}+{ {\mathcal H} }_{dip}+{ {\mathcal H} }_{h}$$

Where ${ {\mathcal H} }_{exc}\,$refers to exchange interaction, ${ {\mathcal H} }_{an}$ represents the total magneto-crystalline anisotropy which in turn can be broken down in different components, $\,{ {\mathcal H} }_{dip}$ stands for magnetic dipolar interactions, and ${ {\mathcal H} }_{h}$ represents the Zeeman interaction due to the influence of a uniforms external magnetic field.

Exchange interaction is shown in Eq. 2, where ${\vec{S}}_{{\rm{i}}}$ are three-dimensional unitary classical Heisenberg spins and ${J}_{exc}$ is the corresponding exchange interaction. The sum runs over each *i* atom interacting with its *j*^*th*^ near neighbors within a cutoff radius of $3\,muc$.

2 $${ {\mathcal H} }_{exc}=-\sum _{i,j}\,{J}_{exc}{\vec{S}}_{i}.{\vec{S}}_{j}$$

with,

3 $${J}_{exc}={J}_{o}\frac{2{k}_{F}{R}_{ij}Cos(2{k}_{F}{R}_{ij})-Sin(2{k}_{F}{R}_{ij})}{{(2{k}_{F}{R}_{ij})}^{4}}$$

The magnitude of ${J}_{exc}$ was calculated as a function of the pair distance ${R}_{ij}$ in the framework of a RKKY approximation^8–11^. Election is based in our interest of considering variations in ion distance. Such election for ${J}_{exc}\,$is supported by several DFT studies in metals where curve of ${J}_{exc}$ vs. distance between magnetic ion presents similar tendencies^12,13^. The length of the Fermi wave vector, ${k}_{F}\,$, was set to 1, which is a typical value for metals^11^. ${J}_{o}$ is a fitting parameter which is chosen depending on the system to be considered. In our case, and for general purposes, this value was fitted in such a way to obtain ${J}_{exc}({R}_{ij}=1\,muc)\,=10\,meV$.

Components of the crystalline anisotropy term are specified in Eq. 4. Three components were considered: cubic magneto-crystalline anisotropy $\,{ {\mathcal H} }_{cryst}$, surface anisotropy $\,{ {\mathcal H} }_{surf}$ and inter-granular boundary anisotropy $\,{ {\mathcal H} }_{boun}$.

4 $${ {\mathcal H} }_{an}={ {\mathcal H} }_{cryst}+{ {\mathcal H} }_{surf}+{ {\mathcal H} }_{boun}$$

It is important to stress that the temperature dependence of the anisotropy has been also considered in attention to experimental works reported^14–16^ as well as the cubic anisotropy distortion on the surface and grain boundaries, where an important local structural disorder is expected. Studies obtained by ab-initio calculations and experimental processes have shown that the electronic configuration of the atoms belonging to these regions is significantly different respect to the extended and homogeneous crystalline medium in the core of the grains^17–20^. Different works have concluded that the magnitude of such an interaction is proportional to the number of surface atoms and small local strains may give rise to an important anisotropy contribution^19,21^.

The ${ {\mathcal H} }_{cryst}$ term of a cubic nature is presented in Eq. 5, where $\,{\alpha }_{i1}$, $\,{\alpha }_{i2}$ and $\,{\alpha }_{i3}$ are the director cosines of the magnetic moments respect to the easy axis of magnetization [100] [001] and [010]. Parameters ${K}_{1i}$ and ${K}_{2i}$ account for the magnitude of the mangetocrystalline anisotropy interaction for which a functional dependence on temperature, in addition to a local structural term, has been proposed. Hence, an effective expression is shown in Eq. 6, where the anisotropy values ${K}_{ni}\,$involve the product between an effective temperature dependence ${K}_{nef}(T)\,$^14,22^ and an effective crystalline disorder dependence ${K}_{ef}({v}_{i}\,)$. The sub-index *n* represents 1 or 2 for ${K}_{1}$ or ${K}_{2}$ respectively and ${v}_{i}\,$is the magnitude of a distortion vector.

5 $${ {\mathcal H} }_{cryst}=\sum _{i}\,{K}_{1i}({\alpha }_{i1}^{2}{\alpha }_{i2}^{2}+{\alpha }_{i2}^{2}{\alpha }_{i3}^{2}+{\alpha }_{i3}^{2}{\alpha }_{i1}^{2})+{K}_{2i}({\alpha }_{i1}^{2}{\alpha }_{i2}^{2}{\alpha }_{i3}^{2})$$

where,

6 $${K}_{ni}={K}_{nef}(T)\,{K}_{ef}({v}_{i}\,)$$

On the other hand, anisotropy decreases rapidly as the temperature approaches the critical temperature^14–16^. On this basis, we propose the relationship given by Eq. 7 accordingly with experimental reports^11,23^. The parameter ${T}_{A}$ refers to an inflexion point generally observed in the curves. ${K}_{no}$ and ${K}_{n\infty }$ represent anisotropy values at zero and high temperature. That values were $\,{K}_{10}=0.05\,meV$, ${K}_{20}=0.02\,meV$ and ${K}_{1\infty }={K}_{2\infty }=0.$ according with typical reported values^14,22^.

7 $${K}_{nef}(T)=A({K}_{no}-{K}_{n\infty })\left(1-\,\tanh \,2\left(\frac{T}{{T}_{A}}-1\right)\right)+{K}_{n\infty }$$

The basis for understanding this surface effect has been exposed by Néel^24^. Where a sum of uniaxial anisotropy contribution per each nearest-neighbor is considered. This approach has been employed for explaining surface effects in nanoparticles^25^ and single clusters^19^. Thus, we propose the introduction of ${K}_{ef}({v}_{i})\,$presented in Eq. 8 in order to account for the loss or lacking of neighbors (or dangling bonds) and local distortions around each atom $i$, which become more relevant at grain boundaries and intergranular regions where the degree of local structural disorder is higher and where the crystal symmetry breaks down. Parameter ${v}_{i}$ is the magnitude of the resulting vector ${\overrightarrow{R}}_{ij}$ of the first neighbors positions which determines the loss of cubic symmetry and it is obtained from Eq. 9.

8 $${K}_{ef}({v}_{i})\,={e}^{-\gamma {v}_{i}}$$

9 $${\overrightarrow{v}}_{i}=\sum _{ < i,j > }{\vec{R}}_{ij}$$

The parameter $\gamma$ can be taken as depending on sample properties. In this study we have set $\gamma$ = 1. The ${\overrightarrow{v}}_{i}$ vector plays an important role for $\,{ {\mathcal H} }_{surf}$ and $\,{ {\mathcal H} }_{boun}$. The magnitude is an indication of the degree of local structural distortion and the resulting direction stands for the single site uniaxial anisotropy direction. A uniaxial surface anisotropy dealing with the surface of the film is suggested in Eq. 10 where an effective surface constant is given by ${K}_{S\_ef}={\varepsilon }_{S}{v}_{i}{K}_{1}$. Uniaxial axis is defined by the unitary vector associated to ${\overrightarrow{v}}_{i}$ and a parameter of proportionality ${\varepsilon }_{S}$ was fixed in 0.2 according to a previous work^26^, which is a measure of the strength of the anisotropy or the spin-orbit coupling.

10 $${ {\mathcal H} }_{surf}=-\,\sum _{i\in Surf}\,{K}_{S\_ef}{({\vec{S}}_{i}\cdot {\hat{v}}_{i})}^{2}$$

In similar way, a uniaxial anisotropy is considered for inter-granular regions. Hamiltonian is represented by Eq. (11) where ${K}_{B\_ef}={\varepsilon }_{B}{v}_{1}^{2}{K}_{1}$. The ${\varepsilon }_{B}$ parameter can be fitted according to experimental properties. In fact, the magneto-elastic energy values are considerably larger than the volume magnetocrystalline anisotropy and they exhibit similar magnitudes to the exchange interaction^27^. As a consequence, small strains may give rise to important magnetic changes^28^. A value of 10 for ${\varepsilon }_{B}$ was used as in previous works^27^, however different values of this parameter can be assumed in order to evaluate the effect of the strength of the anisotropy in the grain boundaries where a high degree of structural distortion is expected.

11 $${ {\mathcal H} }_{boun}=-\,\sum _{i\in Boun}\,{K}_{B\_ef}{({\vec{S}}_{i}\cdot {\hat{v}}_{i})}^{2}$$

Concerning the magnetostatic interactions of nano-granular systems, some models have been proposed for explaining the collective behaviour but none of them has been conclusive^29^. Here, dipolar interactions were also considered by using Cartesian coordinates at two levels. The advantage of Cartesian coordinates was demonstrated by comparison to spherical harmonics in Fast Multipole Method (FMM) and Fast Fourier Transform (FFT) in^30^. The size of each cell was the same of the division employed for parallelization purposes. The dipolar energy is given by:

12 $${ {\mathcal H} }_{dip}=\frac{D}{2}\left[\begin{array}{c}\sum _{i,j\in  {\mathcal L} ,i\ne j}\,\frac{{\vec{S}}_{i}\cdot {\vec{S}}_{j}}{{r}_{ij}^{3}}-3\frac{({\vec{S}}_{i}\cdot {\vec{r}}_{ij})({\vec{S}}_{j}\cdot {\vec{r}}_{ij})}{{r}_{ij}^{5}}\\ +\sum _{i,k\in  {\mathcal R} - {\mathcal L} }\,\frac{{\vec{S}}_{i}\cdot {\vec{S}}_{k}}{{r}_{ik}^{3}}-3\frac{({\vec{S}}_{i}\cdot {\vec{r}}_{ik})({\vec{S}}_{k}\cdot {\vec{r}}_{ik})}{{r}_{ik}^{5}}\end{array}\right]$$

Here, indexes $ij$ and $ik$ refer to interactions between spin-spin and spin-cell moments respectively (near field and far field respectively). First sum is over all spins into a ${\mathcal L}$ region that corresponds to the cell of ${\vec{S}}_{i}$ and the near neighbors cells (7 cells) according with the distance to the centroids of the cells. ${\mathcal R} - {\mathcal L}$ is the region for sums over spin moments of $k$ cell, where ${\vec{S}}_{k}=\sum _{j\in k}{\vec{S}}_{j}$ can be considered as a macrospin. This region considers replicas until a cut-off radius of 5 *L*. The magnitude of ${\vec{r}}_{ik}$ is the distance between ${\vec{S}}_{i}$ and the centre of mass of the $k$ cell. The constant $D$ can be obtained for each particular material from $D={\mu }_{0}{\mu }_{g}^{2}/4\pi {a}^{3}$ where ${\mu }_{0}$ is the magnetic permeability of free space, and ${\mu }_{g}$ the magnetic moment per atom. This parameter was fixed in 0.01 meV.

Finally, the last term in the Hamiltonian (Eq. 1) refers to the Zeeman interaction and it is represented by Eq. 13, where ${\vec{h}}_{ext}$ represents the in-plane external magnetic field.

13 $${ {\mathcal H} }_{h}=-\sum _{i}\,{\vec{S}}_{i}\cdot {\vec{h}}_{ext}$$

**Figure 2:**
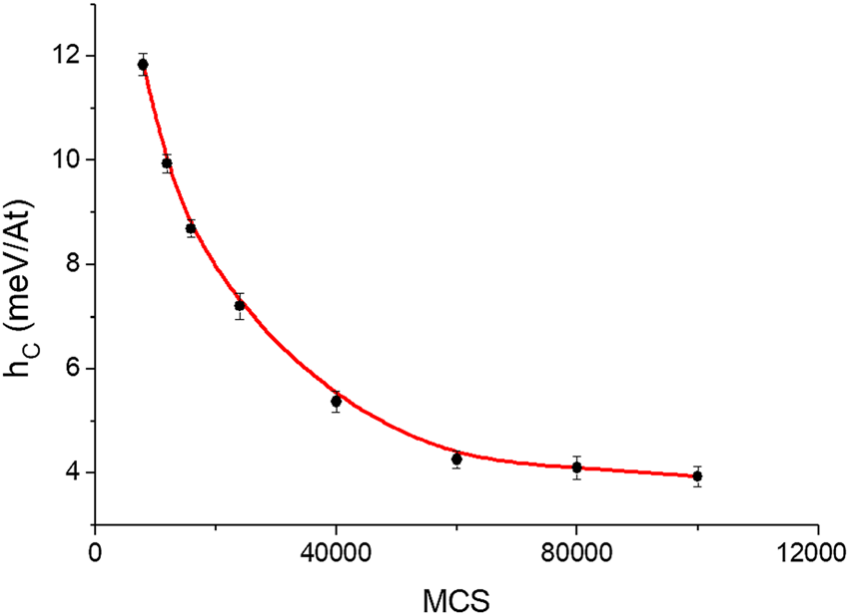
Coercive force as a function of the Monte Carlo Steps at 20 K. The averages were calculated over the last 5 × 10^3^ steps.

For Hysteresis loops simulations, the number of MCS was fixed at 8 × 10^4^ according to the trend of the coercive force $({h}_{C})\,$shown in Fig. 2. Such values are the average over the last 5 × 10^3^ MCS. The external magnetic field step was 0.1 meV. A more detailed analysis of Monte Carlo steps in coercive field of ensemble of single domains particles can be seen in the reference^31^.

It could be thought that the number of parameters selected for the simulation is high. Nevertheless, all of them are essentials to determine the behaviour of these complex systems. Under a reduced set of parameters, previous studies based upon grains size evidenced a range in which correlation between grains is low and phenomena needs to be better studied^32^. Furthermore, based on previous experiences in the simulation area^7,26,32^, it has been concluded that if parameters are adjusted to experimental ranges, how it was done, an analysis of magnetic behaviour of nano-poly-crystalline films with ferromagnetic magnetic cubic cell can be obtained. That is due to the different magnetic contributions acts in ranges of energy well defined. The differences are stablished respect to exchange interaction. Thereby i) magneto-crystalline anisotropy values are found in the µeV range while exchange interactions values are found in the meV range. Then, with grains in nanoscale, anisotropy depends strongly of the loss of correlation by grain size effect more than magneto-crystalline anisotropy. ii) Experimentally, surface anisotropy generated a perpendicular orientation well stablished at very low thickness, less than 10 muc, then that thickness was stablished like criterium iii) dipolar interaction is proportional to *D* parameter obtained by mean $D={\mu }_{0}{\mu }_{g}^{2}/4\pi {a}^{3}$. All these parameters produce dipolar interactions with *D* values in µeV range. This is not a parameter with great local variability because depends mainly on local spin. iv) Just grain boundary anisotropy might have different magnitudes orders and alters significantly the magnetic properties by direct competition with exchange interaction when a loss of grain correlation is caused. Both can be found in the same energy scale. Then boundary parameters were selected based in an interval in which boundary exerts notable influence.

## Results

**Figure 3:**
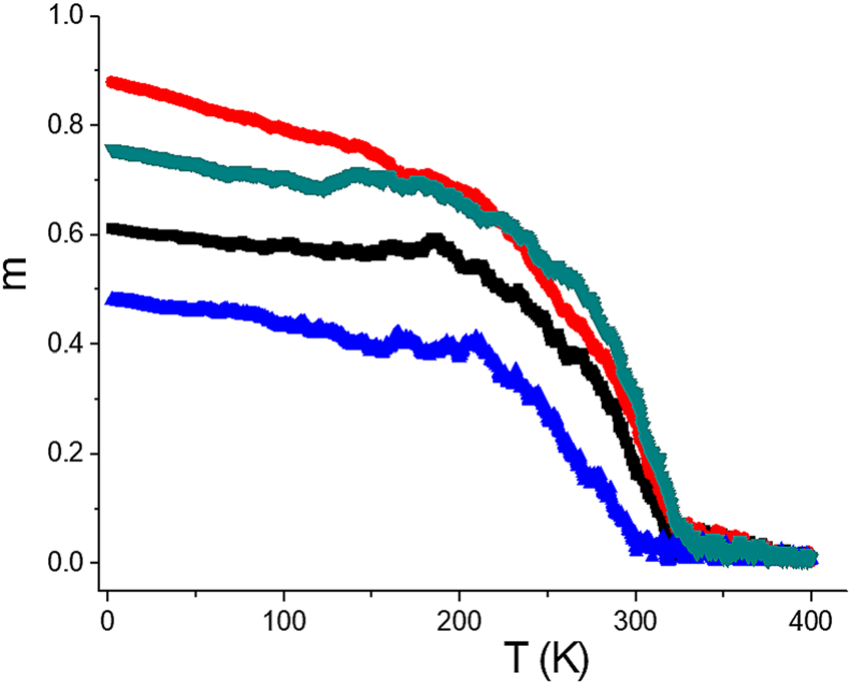
Some examples of different paths of the spontaneous magnetization as a function of temperature. Paths were obtained in cooling processes from different initial random states keeping the same number of grains.

Figure 3 shows four different results of the magnetization cooling process in different samples by keeping the same number of grains but different realizations (different initial random seed numbers). As can be observed, even though transitions are practically at similar temperatures, the low temperature magnetization can be very different. Such a metastability has been reported to occur in nanostructured ferrite samples of Mg_0.95_Mn_0.05_Fe_2_O_4_, where samples having almost an identical particle size distribution can exhibit different spontaneous magnetization values^33^. In that work, authors concluded that differences are strongly influenced by long range interparticle interactions and by local structural disorder giving rise to different realizations of the grain distribution including different crystallite orientations. In our case, as we will demonstrate, local structural disorder can result in zones of the sample, e.g. those with an average coordination number greater than the nominal one, making the short range magnetic coupling between grains a determinant factor for having different spontaneous magnetizations.

**Figure 4:**
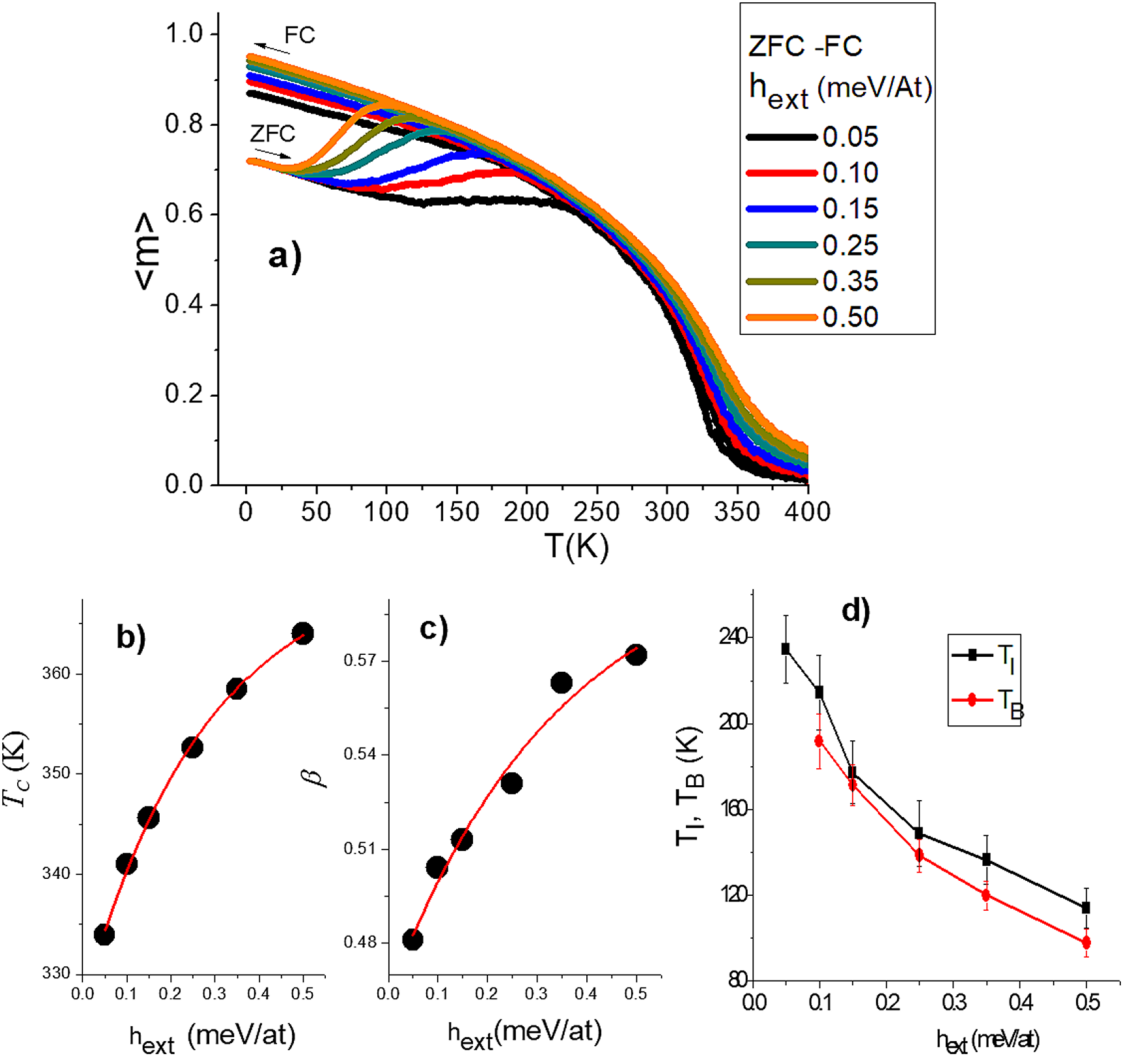
(**a**) ZFC-FC curves for different values of external magnetic field. Dependences with *h*_*ext*_ of the transition temperature and the critical exponent *β* are shown in (**b**) and (**c**) respectively. (**d**) Behavior of the irreversibility and blocking temperatures with the external field.

Zero-field-cooled (ZFC) and field-cooled (FC) curves for different external field values are shown in Fig. 4a. Each curve is the average over 5 simulations. The sample is first cooled without-field until the lowest temperature. After that, an external field is applied while temperature increase, and the magnetization is recorded. This record is denoted as ZFC. Subsequently, the sample reached the highest temperature, data are recorded during cool process to lowest temperature keeping the external field. This record is denoted as FC. As expected, ZFC-FC curves are smoother than those presented in Fig. 3, due to the preferential anisotropy direction of each grain imposed by the external field direction.

The critical exponent $\beta$ associated to the magnetization and the transition temperature ${T}_{C}$ were inferred by fitting the data in a vicinity around ${T}_{C}$ inspired by concept of critical temperature distribution per grain presented by Berger, A^34^. and the following relationship^35^:

14 $$M(T)=A{t}^{\beta }(1+B{t}^{{\beta }^{{\prime} }}+C{t}^{{\beta }^{{\prime\prime} }})$$

where $t=1-T/{T}_{C}$ and A, B, C, ${\beta }^{{\prime} }$ and ${\beta }^{{\prime\prime} }$ are fitting parameters. Results are summarized in Figs. 4b,c respectively as a function of external field. Extrapolation to zero field allowed to obtain $\beta =0.46\pm 0.03$ and $\,{T}_{C}=327.3\pm 2.06$. Our exponent is greater than the one observed in pure and homogeneous 3D classical Heisenberg models having $\beta =0.36$^35^. Such a difference is attributed to local structural characteristics of our system not observed in pure models (e.g. single-crystal films) and consistent with a distribution of critical temperatures.

Figure 4d presents the results of blocking and irreversibility temperatures, ${T}_{B}$ and ${T}_{I}$ respectively. The difference between these temperatures is ascribed to the grain size distribution having different ${T}_{B}\,$^36^. Both curves decrease in a monotonous manner. The behaviour found is in agreement with different experimental results presented by M. Knobel *et al*.^37^ and other reports^38,39^. In the former, authors compare the field dependence of the blocking temperature in a γ-Fe_2_O_3_ monolayer sample and diluted nanoparticles. While for non-interacting nanoparticles a linear behaviour with $\,\vec{h}\,$was observed, for interacting particles the experimental behaviour showed a similar trend to the obtained in this work.

**Figure 5:**
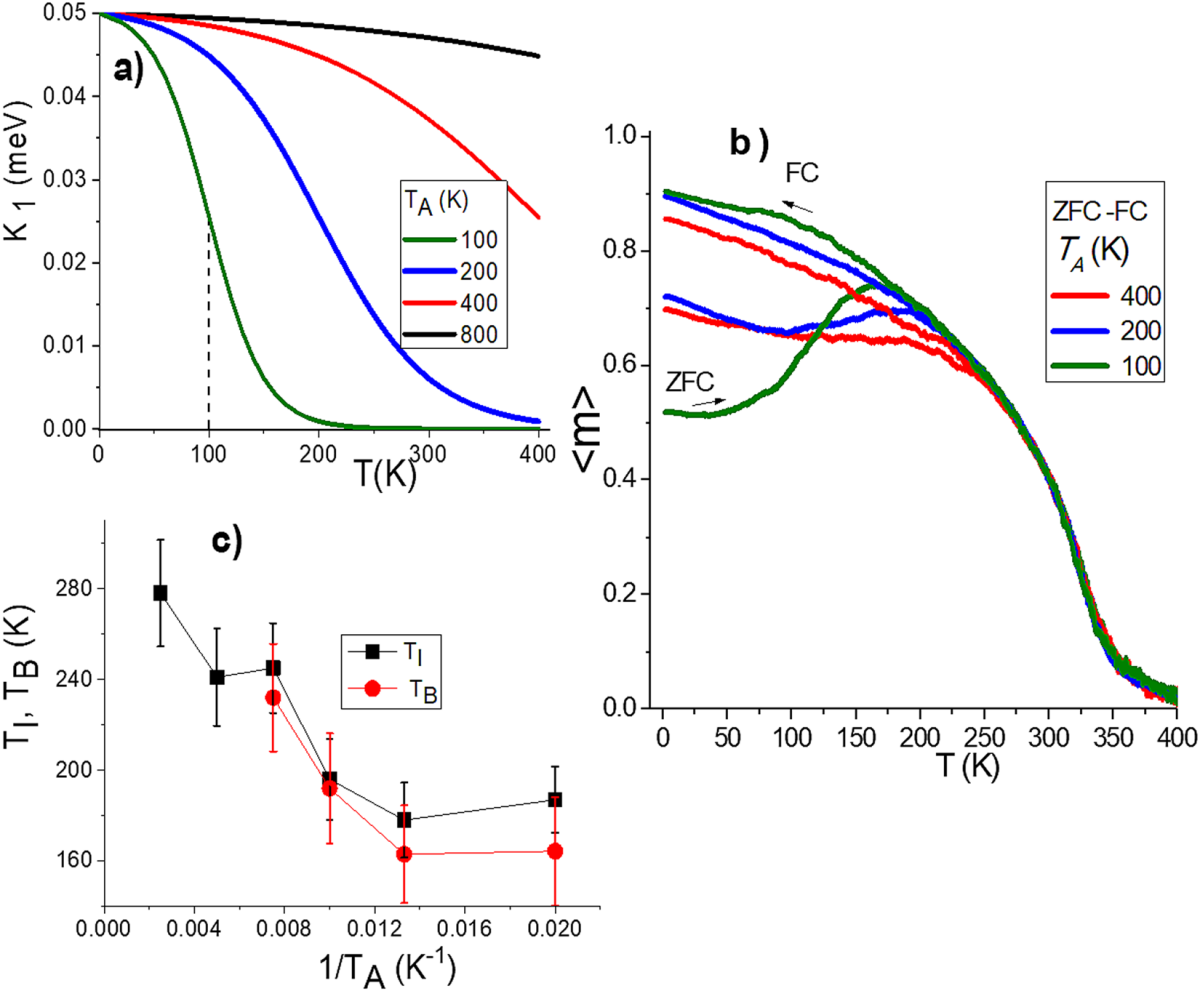
(**a**) Crystalline anisotropy per spin moment as a function of temperature for different values of ${T}_{A}$ according to Eq. (7), the temperature at inflexion point is identified by dash line for ${T}_{A}=100K$, (**b**) Influence of ${T}_{A}$ over three different ZFC-FC curves and (**c**) blocking and irreversibility temperatures presented as function of $\,1/{T}_{A}$.

On the other hand, changes in crystalline anisotropy were implemented to analyse the influence over the blocking temperature. Different magnitudes of ${K}_{1}$ did not show any influence. However, changes of ${T}_{A}$, the temperature at the inflexion point of ${K}_{nef}(T)$ (see Fig. 5a) evidenced an interesting behavior as can be observed in Fig. 5b through the ZFC-FC curves. Initial configurations were obtained from previous cooling processes. As it was already pointed out, in the cooling process spontaneous magnetization can reach different values. Therefore, ZFC curves may eventually overlap at some points. Nevertheless, as it is observed in Fig. 5c, the blocking and irreversibility temperatures exhibit a defined tendency to decrease as a function of $\,1/{T}_{A}$.

**Figure 6:**
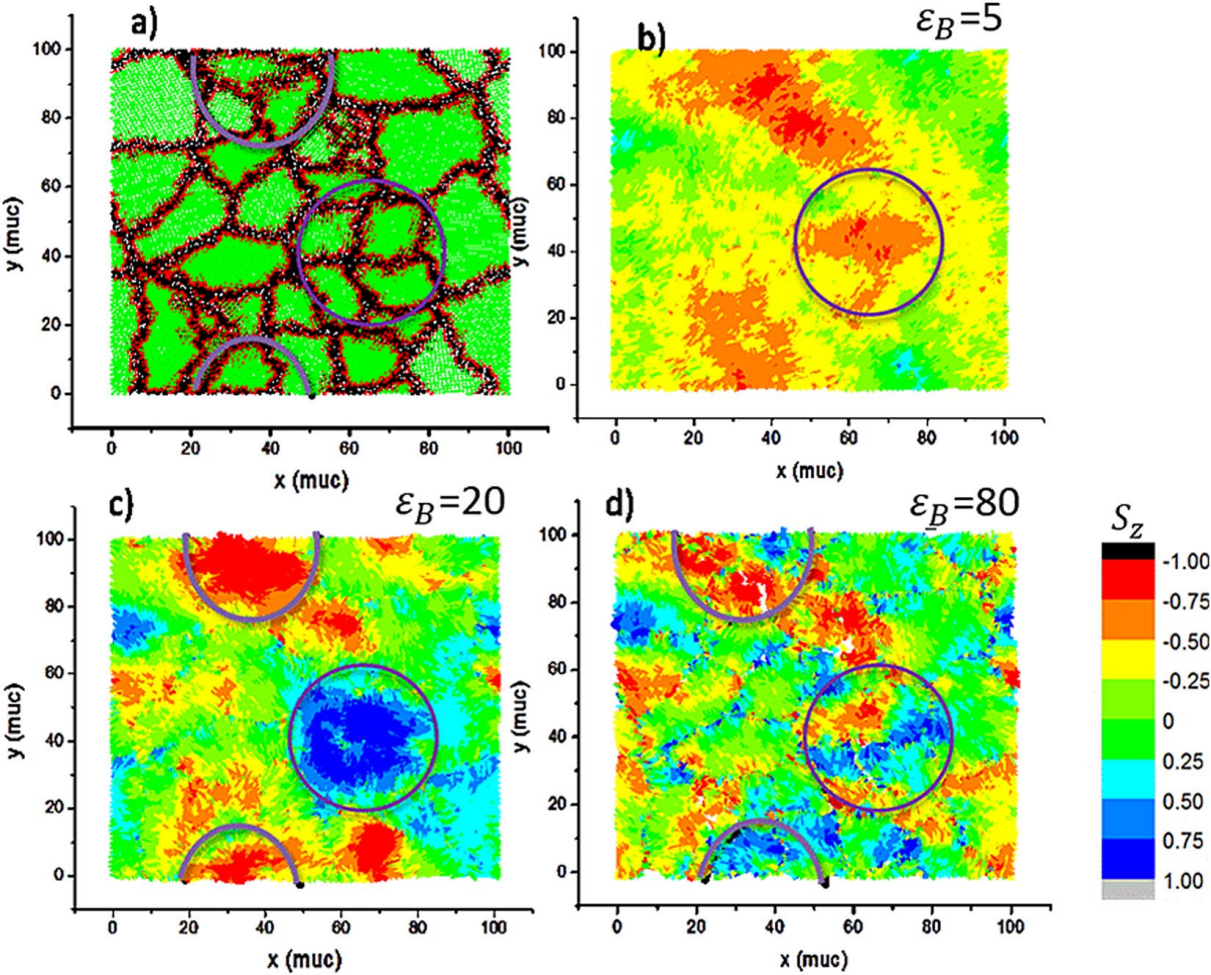
(**a**) Structural distribution of grains for a particular sample, (**b**), (**c**) and (**d**) representations of $\,{S}_{z}$ magnetic components for final states at 2 K in cooling process for ${\varepsilon }_{B}=5$, $\,{\varepsilon }_{B}=20$, and $\,{\varepsilon }_{B}=50$, respectively. Circle evidence a correspondence between grain boundaries and domains at different values of ${\varepsilon }_{B}$.

Figure 6 shows the correlation between the structural landscape and the local ${S}_{z}$ components by means of colormaps representations of a final state in a cooling process without external field and at different values of $\,{\varepsilon }_{B}$. Such values can vary in order to evaluate the effect of the strength of the anisotropy in the boundaries. To do so, several values of the ${\varepsilon }_{B}$ parameter were considered. At low ${\varepsilon }_{B}$ values, the magnetization is more homogenous without sharp changes of the local ${S}_{z}$ components as is presented in Fig. 6b. As ${\varepsilon }_{B}$ increases, a clustering magnetic effect appears as it is possible to observe in Fig. 6c for ${\varepsilon }_{B}$ = 20, where such a domain may involve more than one grain. In contrast and according to Fig. 6d, high values of ${\varepsilon }_{B}$ lead to a boundary disorder effect with an inhomogeneous magnetization. Only big grains can present some reduced domain in their interior. A similar behaviour was observed when considering the other components ${S}_{x}$ and ${S}_{y}$ but with a greater number of domains involved. This is due to the preferential *x-y* given by dipolar interaction and the symmetry of the system.

**Figure 7:**
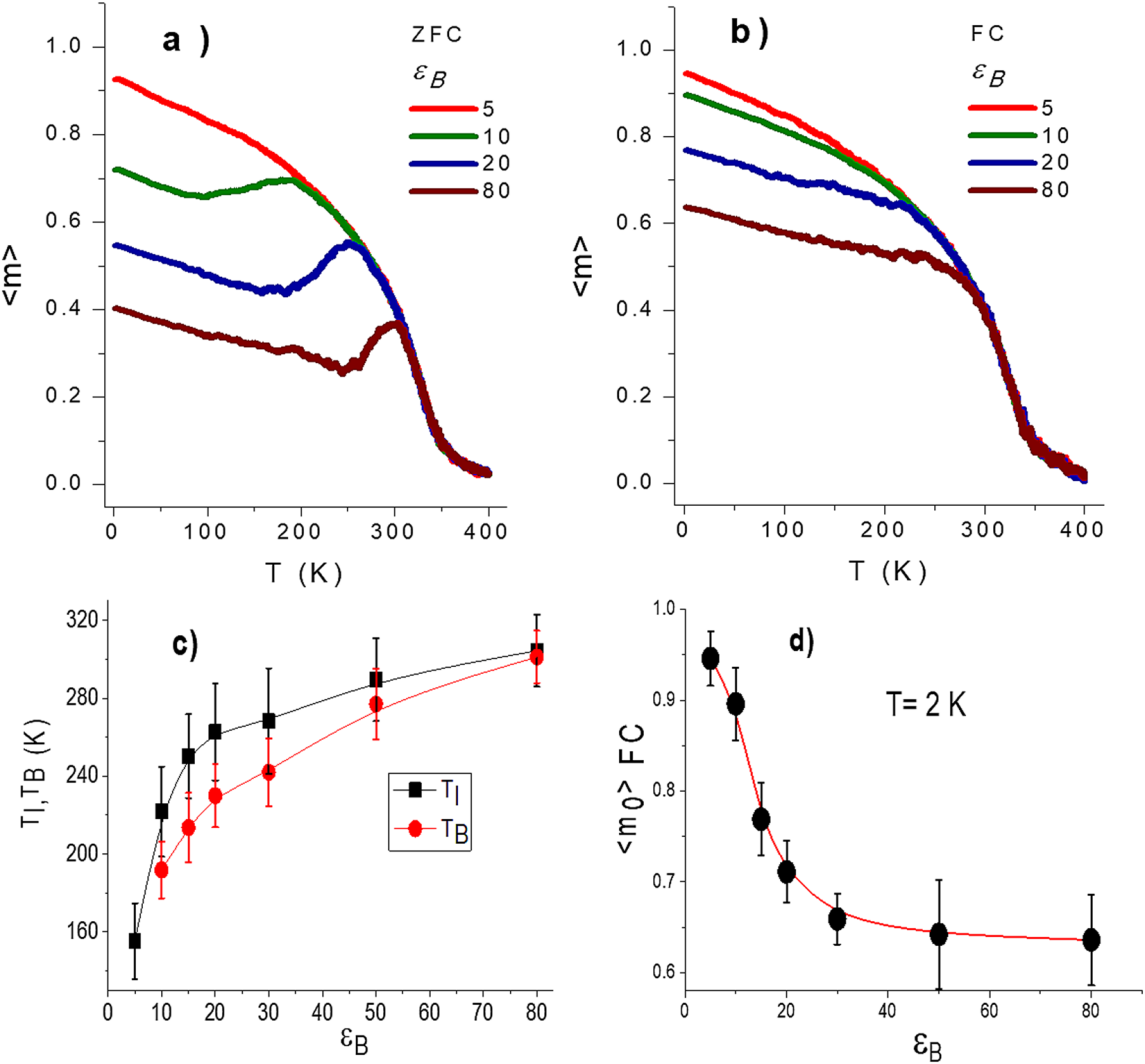
Influence of the strength of the boundary anisotropy ${\varepsilon }_{B}$ on the ZFC and FC curves is shown in (**a**) and (**b**) respectively. (**c**) Blocking and irreversibility temperatures as a function of $\,{\varepsilon }_{B}$. (**d**) Low-temperature dependence of the magnetization with $\,{\varepsilon }_{B}$ in FC.

Figures 7a,b show the effect of the boundary anisotropy parameter $\,{\varepsilon }_{B}$ upon the shape of the ZFC-FC curves respectively. These curves are the average over five simulations. The respective blocking and irreversibility temperatures are presented in Fig. 7c as a fuction of the ${\varepsilon }_{B}$ parameter. An increment in both quantities is observed. When increments in the boundary anisotropy are introduced, domains turn out magnetically harder giving rise to ha higher barrier to be overcome and therefore a greater blocking temperature is needed. It is interesting to remark however, that contrary to the well-known linear relationship of the blocking temperature with the density of anisotropic energy for a given volume, here, two different linear regimes seem to be observed, one below ${\varepsilon }_{B}=20$ and the other one at higher values. According to the results presented in Fig. 6, such value of ${\varepsilon }_{B}\,$corresponds to the limit above which grain boundaries coincide with the domain limits. The ${m}_{oFC}$, the magnetization at 2 K in field cooling is presented as a function of ${\varepsilon }_{B}\,$ parameter in Fig. 7d. According to this figure, high values of ${\varepsilon }_{B}\,$ make that the disorder effect produced at the borders destroys homogeneous magnetization within the grains. Therefore ${T}_{B}$, $\,{T}_{I}$ and ${m}_{oFC}\,$ response to this variations of ${\varepsilon }_{B}$, where at low values of ${\varepsilon }_{B}\,$ the pinning of the magnetic moments in the domains depends on the collective behaviour of the film, at intermediate values of ${\varepsilon }_{B}\,$ the insulating effect of the borders makes the domains become independent in each grain, and at a high value of ${\varepsilon }_{B}\,$ that the film behaves similar to a pin glass system, in which the coupling between the magnetic moments shows a strong degree of frustration.

**Figure 8:**
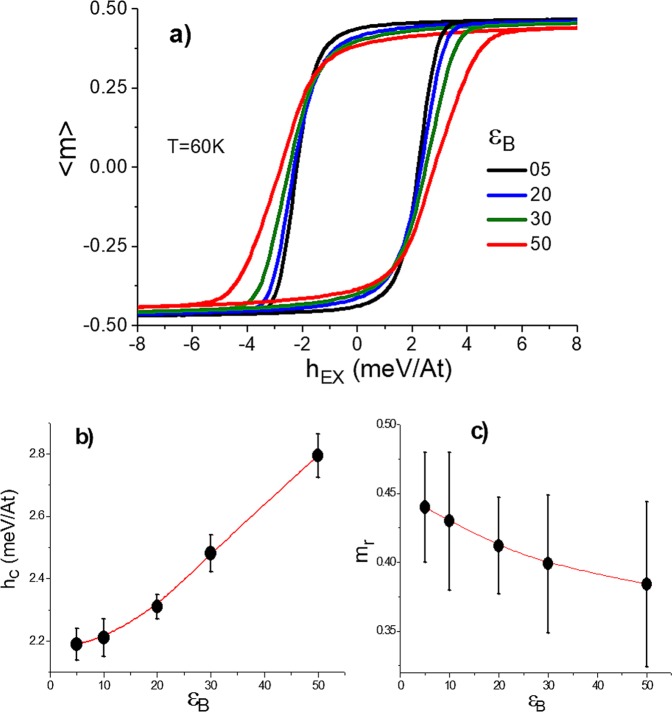
(**a**) Hystesis loops at different values of $\,{\varepsilon }_{B}$. (**b**) and (**c**) coercive field and remanence as a function of $\,{\varepsilon }_{B}$, respectively. Increments in anisotropy strength promote a remarkable coercive force increase.

Regarding the hysteretic properties, hysteresis loops were also simulated and they are shown in Fig. 8a for different values of ${\varepsilon }_{B}\,$ at a temperature below the critical temperature. As can be observed M-H loops the system becomes magnetically harder as the parameter ${\varepsilon }_{B}$ increases, which in turn makes the coercive force to increase as it is shown in Fig. 8b, whereas the remanence tends to slightly diminish (see Fig. 8c). Nevertheless, it is worth noting that despite of varying the boundary anisotropy, no humps in the hysteresis loops are observed, which means that the mechanism for magnetization reversal takes place in a gradual way where the system behaves as a whole and not in a differentiated fashion implying separated contributions of the grain cores and grain boundaries. Different hysteresis loop simulations varying ${T}_{A}$ and ${K}_{1}$ do not exert influence upon the coercive force and remanence. That is due to exchange interaction which is 200 times magnitude orders greater than crystalline anisotropy.

## Conclusions

The interplay of the temperature dependence of the grain boundary anisotropy and local structural disorder in nanostructured thin films was analyzed. Results revealed that i) by keeping the same number of grains, different realizations gave rise to different spontaneous magnetizations, ii) the critical exponent of the magnetization was different from that of pure models. The difference was attributed to the complexity of the lattice structure in accordance with the distribution of critical temperatures found in other reports of inhomogeneous films iii) the way in which the boundary anisotropy varies with temperature and its strength were determinant factors for blocking temperatures, and iv) hysteresis loops below critical temperature were characterized by a high degree of symmetry with a coherent mechanism of reversal rotation, without humps or jumps.

## Data Availability

The datasets generated during and/or analysed during the current study are available from the corresponding author on reasonable request. OriginPro 8 software was used for image processing^40^.
